# Insights into the Complement System of Tunicates: C3a/C5aR of the Colonial Ascidian *Botryllus schlosseri*

**DOI:** 10.3390/biology9090263

**Published:** 2020-09-01

**Authors:** Anna Peronato, Nicola Franchi, Loriano Ballarin

**Affiliations:** Department of Biology, University of Padova, 35121 Padova, Italy; anna.peronato@studenti.unipd.it (A.P.); loriano.ballarin@unipd.it (L.B.)

**Keywords:** C3a/C5aR, *Botryllus*, tunicates, complement

## Abstract

As an evolutionary ancient component of the metazoan immune defense toolkit, the complement system can modulate cells and humoral responses of both innate and (in jawed vertebrates) adaptive immunity. All the three known complement-activation pathways converge on the cleavage of C3 to C3a and C3b. The anaphylatoxin C3a behaves as a chemokine in inflammatory responses, whereas C3b exerts an opsonic role and, ultimately, can activate the lytic pathway. C3aR, one of the mammalian receptors for C3a, is a member of the G-protein-coupled receptor family sharing seven transmembrane alpha helixes. C3aR can act as a chemokine and recruit neutrophils, triggering degranulation and respiratory burst, which initiates an inflammatory reaction. Mining the transcriptome of the colonial ascidian *Botryllus schlosseri*, we identified a transcript showing homology with both mammalian C3aR and C5aR. The gene (*bsc3/c5ar*) is actively transcribed in morula cells, the circulating immunocyte triggering the inflammatory reactions in response to the recognition of nonself. Its transcription is modulated during the recurrent cycles of asexual reproduction known as blastogenetic cycles. Moreover, the treatment of hemocytes with C3aR agonist, induces a significant increase in the transcription of BsC3, revealing the presence of an autocrine feedback system able to modulate the expression of C3 in order to obtain a rapid clearance of potentially dangerous nonself cells or particles. The obtained results support the previously proposed role of complement as one of the main humoral components of the immune response in tunicates and stress the importance of morula cells in botryllid ascidian innate immunity.

## 1. Introduction

Inflammation is one of the first lines of defense in the innate immune system of metazoans. It involves the recruitment of immunocytes to the infection sites, their extravasation, and consequent degranulation with the induction of cytotoxicity [1]. The inflammatory events operate in tight association with the complement system (CS), one of the most ancient immune modulators of metazoans [2]. 

Once activated, the CS leads to the cleavage of the core protein C3 into component proteins C3a and C3b [2,3]. The anaphylatoxin C3a, which shares many functional behaviors with the closely related molecules C4a and C5a, plays a central role in inflammatory response. C3a recruits and activates immunocytes, triggers degranulation, induces phagocytosis, and associated respiratory burst and reactive oxygen species (ROS) production. C3a works primarily by interacting with its receptor, known as C3a receptor (C3aR) [4,5,6].

C3aR is a member of the G protein-coupled receptor family; all members of this family have seven transmembrane (7TM) alpha helixes [7]. This receptor has been widely described and characterized in mammals [8], where it is expressed in various immunocytes (e.g., monocytes, dendritic cells, neutrophils, basophils) [9]. Mammalian C3aRs feature a large extracellular loop (ECL2) between the fourth and fifth transmembrane regions (TM4 and TM5) [7] that is required for the high-affinity binding of C3a [10]. As for non-mammalian vertebrates, up to now, C3aR has been described in amphibians and teleost fish [11], although avian and reptilian C3aR gene sequences are present in databases. 

Molecular data indicate that vertebrate C3aRs and C5aRs (the receptors for C5a) derive from a duplication event from a common ancestor. Since teleost fish possess both C3aR and C5aR, this suggests that the duplication occurred before the emergence of bony fish [8]. 

In invertebrate chordates, functional C3a anaphylatoxin with chemotactic properties has been described in the cephalochordate *Branchiostoma japonicum* [12] and the tunicate *Ciona intestinalis* [13]. In *Ciona*, a chemotactic receptor was also identified and characterized [14]. 

*Botryllus schlosseri* is a cosmopolitan compound ascidian, and a member of the tunicates, a group of animals representing the closest relatives to vertebrates. It is one of the most used organisms for the study of tunicate immunobiology and immune system evolution [15]. A colony includes three blastogenetic generations: actively filter-feeding zooids, primary buds emerging from the zooid body wall, and secondary buds on primary ones. Through cyclical generation changes or take-overs (TOs), the colony periodically rejuvenates: during this period (lasting 24–36 h), old zooids lose their functionality, close their siphons, and are progressively resorbed. In the meantime, buds grow to adult size and replace old zooids whereas budlets become buds able to give rise to a new budlet generation. The period of time between two successive TOs is defined as a colony blastogenetic cycle [16]. 

In this species, we already identified some complement components such as C3 (BsC3), FactorB (BsfB), ficolin (Bsficolin), MASP (BsMASP) [17]. In particular, BsC3 shares with other metazoan C3s the presence of a conserved cleavage site that generates C3a and C3b [17]. We already studied some of the interactions between the complement system and immunocytes, demonstrating the involvement of complement C3, likely through C3b, in phagocytosis, as suggested by its reduction when C3 activation is blocked [17,18]. However, no data on the possible involvement of C3a in *Botryllus* immunity has been reported up to now. In order to fill this gap and acquire new information on ascidian complement, in the present work, we mined our *Botryllus* transcriptome [18] looking for sequences with similarity to human C3aR. 

## 2. Material and Methods

### 2.1. Animals

Colonies of *B. schlosseri* were collected in the Southern part of the Lagoon of Venice and left to adhere to glass slides in aquaria at 18 °C for 5 days. Animals were fed daily with Phyto Marine (Oceanlife, Bologna, Italy) and living *Tetraselmis chuii* cells. Each *B. schlosseri* colony derives from a single tadpole-like larva that metamorphoses into an oozoid, the colony founder. The colony increases its size through several cycles of asexual reproduction (blastogenetic cycles). The filtering adults are organized in star-shaped systems of 10–15 zooids, each with its own oral siphon; in the center of each system, a common cloacal siphon opens to the exterior. Each blastogenetic cycle lasts about one week at 20 °C and begins with the appearance of thickenings of the zooid body wall (bud primordia): we called this phase, lasting one day, early cycle (EC). The blastogenetic cycles end with the TO, lasting 24–36 h [16]. The colonial developmental phases lying more than one day from the TO [16,19] are collectively called mid-cycle (MC). A common circulatory system, in the form of a vessel network within the common tunic, connects zooids, buds, and budlets [20,21]. Among the circulating hemocytes, immunocytes, i.e., the effectors of immune reactions, are represented by phagocytes and cytotoxic granular (morula) cells [22] and constitute the majority of cells [23]. Phagocytes collectively account for 20–40% of the total circulating cells and include spreading and round phagocytes, the former being actively moving cells that, upon the ingestion of foreign particles or cells, acquire the round morphology. Morula cells are the most abundant hemocyte type (40–60% of total circulating hemocytes [22]) and are the first cells perceiving the presence of nonself [23,24].

### 2.2. Hemocyte Collection

To collect hemocytes, the marginal vessel of colonies (previously immersed in 0.38% Na-citrate in filtered seawater (FSW) to prevent cell clumping) was punctured with a fine tungsten needle, and cells were collected with a glass micropipette. They were then pelleted at 800× g for 10 min and re-suspended in FSW to a final concentration of 5 × 10^5^ cells/mL.

### 2.3. BsC3a/C5aR Sequence Characterization and Phylogenetic Analysis

Mining our *B. schlosseri* EST collection (http://botryllus.cribi.unipd.it; last access: 12 March 2020) [18] and the database of the *B. schlosseri* genome (http://botryllus.stanford.edu/botryllusgenome/last access: 23 July 2020), we identified through BLASTn analysis using the predicted *C. intestinalis* C3aR sequence (Genbank CAI84650.1) the sequence of a transcript with similarity to the vertebrate transcripts for C3aR, referred to as BsC3a/C5aR. The 3’ rapid amplification of the cDNA ends (RACE) was performed using the 2nd Generation of the 5’/3’ RACE Kit (Roche Diagnostics, Basel, Switzerland). In order to obtain the 3’ sequences of BsC3a/C5aR cDNA, specific primers, reported in Table S1, were designed for nested PCR with anchor reverse primer according to the manufacturer’s instruction. 

Analysis of the gene sequence organization was carried out using SPLIGN (https://www.ncbi.nlm.nih.gov/sutils/splign/splign.cgi?textpage=online&level=form [25]; last access: 14 July 2020).

We used SMART [26] (http://smart.emblheidelberg.de; last access: 5 August 2020) and Clustal Omega [27] (http://www.ebi.ac.uk/Tools/msa/clustalo; last access: 5 August 2020) for multiple alignments using the sequences listed in Table S2, to investigate the architecture domain organization of BsC3a/C5aR. 

For the phylogenetic analysis, alignments were performed with MUSCLE software [28] and assessed using molecular evolutionary genetics analyses (MEGA) version 7 program [29]. We evaluated different amino acid substitution models using MEGA7 and we found that the JTT+G+F was the best fit for our dataset with the lowest Akaike information criterion (corrected AIC scores) = 42,837.9963 and maximum likelihood value (lnL) = −21274.8895. The maximum likelihood (ML) [30] method was used to build phylogenetic trees with the MEGA 7 software [29]. The non-distance-based phylogeny reconstruction neighbor-joining (NJ) [31] and the maximum parsimony (MP) methods were also used to build phylogenetic trees with the MEGA 7 software [29]. The nonparametric bootstrap test [32], with 10,000 replicates, was used to assess the robustness of the tree topologies. Sequences used for phylogenetic analysis included C3aRs and C5aRs from vertebrates and sequences ascribed to C3aR from invertebrate chordates, found in Genbank and Aniseed. Outgroup sequences were obtained from Boshra et al. [11]. They are all reported in Table S2. 

### 2.4. Primer Design, RNA Extraction, cDNA Synthesis, Cloning and Sequencing 

The RNA NucleoSpin RNA XS (Macherey–Nagel, Düren, Germany) kit was used to isolate total RNA from colonies of *B. schlosseri* and its quality was determined by the A_260/280_ ratio and the quality of RNA was determined by the visualization of rRNAs in Midori green (Nippon genetics)-stained 1.5% agarose gels. The first strand of cDNA was reverse transcribed from 1 µg of total RNA at 42 °C for 1 h in a 20 µL reaction mixture containing 1 µL of ImPromII Reverse Transcriptase (Promega, Madison, WI, USA) and 0.5 µg oligo(dT)-Anchor primer or random primers (Promega, Madison, WI, USA).

The primers reported in Table S1 were used for PCR reactions in a 25-µL reaction volume containing 100 ng of cDNA from *B. schlosseri* colonies, 2.5 µL of 10× incubation buffer (PCRBIO Classic Taq, PCR BIOSYSTEMS, London, UK) with 15 mM MgCl_2_, 0.25 µM of each primer, 10 mM of each of the deoxynucleotide triphosphates, and 2 units of Taq polymerase. PCR was performed on a MyCycler (BioRad; Hercules, CA, USA) thermocycler with the following conditions: 94 °C for 2 min, then 40 cycles of 94 °C for 30 s, 55–60 °C for 30 s, 72 °C for 40 s, and 72 °C for 10 min. Amplicons were separated by electrophoresis on 1.5% agarose gel and the corresponding bands were purified with ULTRAPrep Agarose Gel Extraction Mini Prep kit (AHN Biotechnologie, Nordhausen, Germany), ligated in pGEM-T Easy Vector (Promega, Madison, WI, USA), and cloned in DH-5α *Escherichia coli* cells. Positively screened clones were Sanger sequenced at Eurofins Genomics (Ebersberg, Germany) on an ABI 3730XL Applied Biosystems apparatus (Life Technologies Europe BV, Monza, Italy).

### 2.5. Quantitative Real-Time PCT (qRTPCR)

Three laboratory colonies were split into three subclones, 2–3 systems each, and, after 5 days of acclimation, their blastogenetic cycle was followed under the dissection microscope. When at EC, MC, and TO, colonies were collected and their mRNA was extracted as already reported. qRT-PCR was carried out according to the method reported in Franchi and Ballarin [17] to estimate the total amount of mRNA for BsC3a/C5aR. In this case, EC was considered as a reference control. Forward and reverse specific primers for the above-reported transcripts and for the elongation factor 1α (EF1α) were designed and reported in Table S1. All the designed primers contained parts of contiguous exons to exclude contamination by genomic DNA; a qualitative PCR was also carried out before qRT-PCR. In addition, the analysis of the qRT-PCR dissociation curve gave no indications of the presence of contaminating DNA.

The following cycling parameters were used: 3 min at 95 °C (denaturation), 20 s at 95 °C plus 1 min at 60 °C, 45 times (annealing), 15 s at 95 °C, 1 min at 60 °C, 15 s at 95 °C, 15 s at 60 °C (extension). Each set of samples was run three times on an Applied Biosystem 7900 HT Fast Real-Time PCR System (Life Technologies Europe BV, Monza, Italy) and each plate contained cDNA from three different biological samples (*n* = 3) and negative controls. The 2^−ΔΔCT^ method [33] was used to estimate the total amount of mRNA. The amounts of transcripts in different conditions were normalized to EF1α to compensate for variations in the amounts of cDNA.

### 2.6. In Situ Hybridization (ISH)

Using the primers reported in Table S1, as previously described [17], we produced the biotin-labeled antisense riboprobes for BsC3a/C5aR. Hemocytes were collected as previously described and left to adhere for 30 min on SuperFrost Plus (Menzel–Glaser, Braunschweig, Germany) glass slides. Cells were then incubated for 1 h in FSW in the presence or in the absence (control) of either zymosan (1 mg/mL) or *Bacillus clausii* (4 × 10^5^ cells/mL). They were then washed in FSW and fixed for 30 min in a solution of 4% paraformaldehyde plus 0.1% glutaraldehyde in 0.4 M cacodylate buffer, containing 1.7% NaCl and 1% sucrose, at 4 °C. After their permeabilization in a solution of 0.1% Triton X in phosphate-buffered saline (PBS: 1.37 M NaCl, 0.03 M KCl, 0.015 M KH_2_PO_4_, 0.065 M Na_2_HPO_4_, pH 7.2) for 5 min, hemocytes were washed in PBS and preincubated in Hybridisation Cocktail 50% formamide (Amresco, Solon, OH, USA) for 1 h at 55 °C, and hybridized in the same solution containing 1 µg/mL riboprobe, overnight, at the same temperature reported above. They were then washed in SSC (0.3 M NaCl, 40 mM sodium citrate, pH 4.5) for 5 min, and in a solution of 50% formamide in SSC at 55 °C for 30 min followed by an additional washing in PBS containing 0.1% Tween 20 (PBST) at room temperature, for 5 min. Hemocyte monolayers were then incubated in 1% powdered milk in PBST for 1 h (to reduce aspecific staining), in 5% methanol for 30 min (to block endogenous peroxidases), and in Vectastain ABC (Vector) in PBS for 30 min. Finally, cells were incubated in 0.025% 3,3′-diaminobenzidine and 0.004% H_2_O_2_ in PBS for 15 min. Slides were washed in distilled water and mounted in Eukitt (Electron Microscopy Sciences, Hatfield, PA, USA) before cell observation under the light microscope (LM).

### 2.7. Effects of C3aR Agonist

In another experimental series, to investigate the relationship between BsC3a/C5aR activation and BsC3 transcription, three colonies were divided into two subclones and, after an acclimation period of 5 days, the marginal vessels of the subclones, when at MC, were injected with 5 µL of C3aR agonist (Santa Cruz Biotechnology, Dallas, TX, USA) at the concentration of 0.3 µM in DMSO or with the same amount of DMSO in controls. After 24 h, mRNA was extracted from treated and control colonies according to the protocol described above, reverse transcribed to cDNA, and the expression of *bsc3* and *bsc3a/c5ar* was followed by qRT PCR. EF1α was used as a housekeeping gene due to its stable expression. 

### 2.8. Statistical Analysis

Data are expressed as mean ± SD. qPCR experiments were replicated three times (n = 3) with three independent samples. Statistical analyses were performed with the PRIMER statistical program. One-way ANOVA was followed by the Student–Newman–Keuls test to assess significant differences with respect to either EC, in the case of blastogenetic cycle analysis, or colonies injected with DMSO in FSW, in the case of injection experiments. In ISH experiments, at least 200 cells were counted under the LM, in 10 optical fields, at 1250×. Data were compared with the χ^2^ test. 

## 3. Results

### 3.1. An Orthologue of C3aR is Present in the B. schlosseri Transcriptome

By BLAST analysis of our *B. schlosseri* EST collection and 3’ RACE, we identified a transcript with similarity to the mammalian transcript for C3aR and C5aR, that was named BsC3aR/C5aR on the basis of phylogenetic analysis. The sequence was deposited in Genbank under the accession number MN053062 and was confirmed by alignment with the *B. schlosseri* genome. The transcript sequence (g020950) is reported in Figure S1. BLASTn search for the chromosome location in the Aniseed database indicates that it is not located in chromosomes 1–13, but in the residual unresolved scaffold containing the sequences of chromosomes 14–16. 

**Figure 1:**
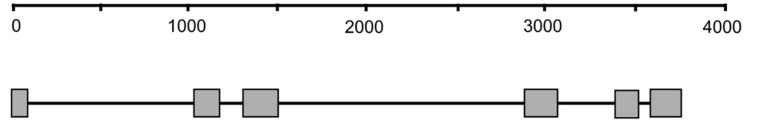
Schematic organization of BsC3a/C5aR gene. Exons are represented by boxes and introns by lines. Numbers refer to the length of the nucleotide sequence.

The gene for BsC3a/C5aR includes 6 exons (Figure 1 and Table 1) with the start and stop codons located in the first and in the last exons, respectively. All the introns are provided with the canonical GT and AG splicing signal consensus (Figure S1) and its transcript has an ORF of 1296 bp that, once translated in silico, results in a protein of 431 amino acids with a putative molecular weight (MW) of 49.3 kDa.

### 3.2. Phylogenetic Analysis

**Figure 2:**
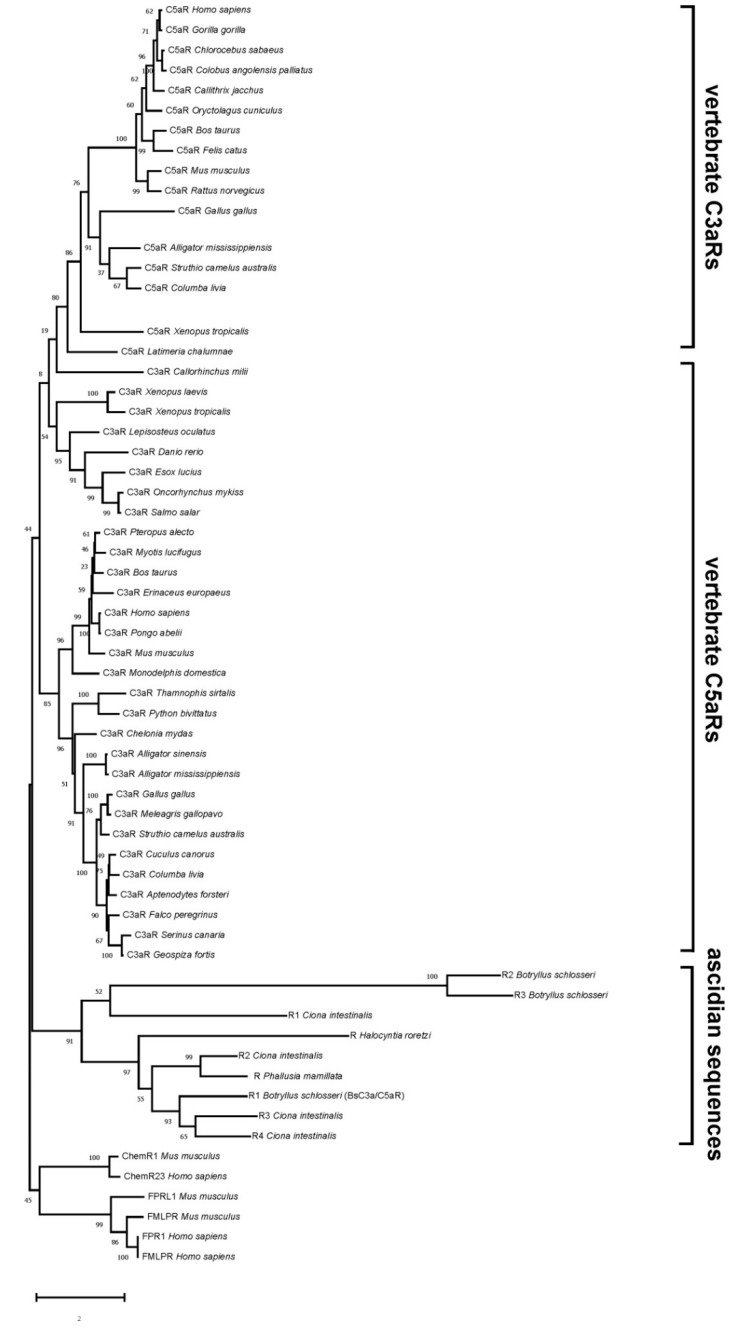
Evolutionary relationships among deuterostome anaphylatoxin receptors obtained with the maximum likelihood (ML) method. Similar topologies were obtained with neighbor joining (NJ) and unweighted pair group method with arithmetic mean (UPGMA). Bootstrap confidence values are indicated at the left of each branch. Sequence accession numbers are reported in Table S2.

The phylogenetic trees obtained with the ML method for C3a and C5a receptors are presented in Figure 2. In addition to the *Ciona* sequence reported by Melillo et al. [14] and our *Botryllus* sequence (reported in the phylogenetic tree and in the multi-alignment as R1 *Ciona intestinalis* and R1 *Botryllus schlosseri*, respectively), we inserted also additional sequences from ascidians found in Genbank and Aniseed. Since a deeper insight revealed that many of them are only short partial sequences, we used only full sequences containing complete open reading frames. In particular, we included amino acid sequences for putative C3a/C5aRs from *B. schlosseri* (R2 and R3, *Botryllus schlosseri*), *C. intestinalis* (R2, R3, R4, *Ciona intestinalis*), *H. roretzi* (R, *Halocynthia roretzi*), and *P. mamillata* (R, *Phallusia mamillata*). See Table S2 for the annotation IDs. To our knowledge, no data on the presence of C3a/C5aRs outside chordates are present in the literature. 

The resulting tree shows the presence of three main clusters, represented by vertebrate C5aRs, vertebrate C3aRs, and ascidian sequences, respectively. All the ascidian sequences cluster together: within this group. *B. schlosseri* R2 and R3 cluster together and have *C. intestinalis* R1 as sister group; the cluster represented by *B. schlosseri* R2 and R3 and *C. intestinalis* R1 diverge from the one represented by the remaining sequences (Figure 2).

The divergence of *Botryllus* R2 and R3 receptors from the rest of the ascidian sequences is likely the consequence of the lack of the large second extracellular loop and the presence of only six, instead of seven TM domains. 

### 3.3. Sequence Aligments

A closer analysis of the ascidian sequences revealed that R2 from *C. intestinalis* and R from *P. mamillata* do not belong to the G protein-coupled receptor family as they lack the typical 7TM domains, having only five and six TM domains, respectively. Analogously, as reported above, the *Botryllus* R2 and R3 receptors have only six TM domains and lack the large extracellular loop. This renders their inclusion within the C3a/C5a receptors highly debatable, and they were excluded from the multi-alignment reported in Figure S2, using the sequence of human C3aR as a reference sequence. 

From the analysis of the multi-alignment, it results that all the ascidian sequences contain the large second extracellular loop, rich in negatively charged Asp and Glu residues, between the 4th and the 5th TM domains that characterize most of the C3aRs and required for proper ligand binding. Various conserved residues are present in the TM domains. The Tyr corresponding to the position 174 of the human sequence is conserved in *Botryllus* R1, *Ciona* R3, and R4, but is lacking in *Ciona* R1 and *Halocyntia* sequences. The two cytosolic Thr residues, corresponding to the positions 463 and 466 of the human sequence, are also conserved in *Botryllus* R1 and *Ciona* R3 and R4; the *Halocynthia* and the *Ciona* R1 sequences have only one and none Thr residues, respectively. 

**Figure 3:**
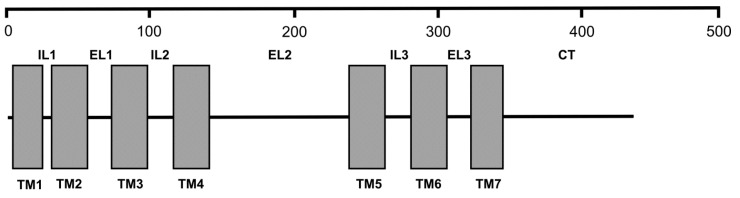
Schematic domain organization of BsC3a/C5aR protein. Numbers refer to the length of the amino acid sequence. IL: intracellular loop; EL: extracellular loop; CT: cytoplasmic tail; TM: transmembrane domain.

Collectively, these data indicate that *Botryllus* C3a/C5aR is a new member of the G-protein-coupled receptor family. Its domain organization is reported in Figure 3.

### 3.4. The Transcription of bsc3a/c5ar Changes during the Colonial Blastogenetic Cycle 

**Figure 4:**
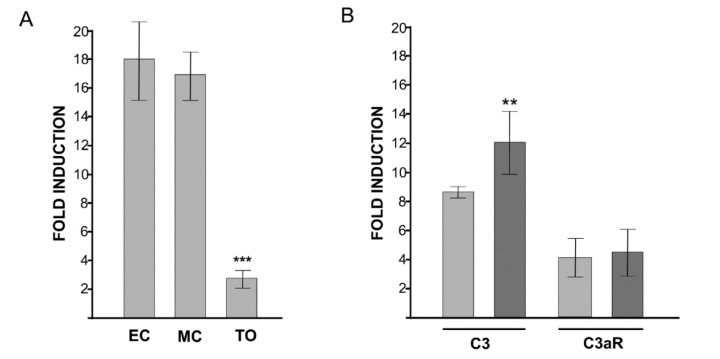
(**A**): level of *bsc3a/c5ar* transcription product in three phases of the colonial blastogenetic cycle as assessed by qRT PCR. EC: early cycle; MC: mid cycle; TO: takeover. (**B**): transcription of *bsc3a/c5ar* and *bsc3* in colonies previously injected with C3aR agonist (dark grey bars) as compared to controls (light grey bars). Asterisks mark significant differences with respect to EC or the control (**: *p* < 0.01; ***: *p* < 0.001).

The transcription level of *bsc3a/c5ar* at EC and MC did not significantly differ; it significantly (*p* < 0.001) decreased to around 1/6 of the above values at TO (Figure 4A). 

### 3.5. C3aR Agonist Affects C3 Transcription

A significant (*p* < 0.05) increase in the transcription of *bsc3* was observed in colonies injected with C3aR agonist. No effects were observed in the extent of transcription of *bsc3ar* (Figure 4B).

### 3.6. Morula Cells are the only Hemocytes Expressing BsC3aR/C5aR mRNA

**Figure 5:**
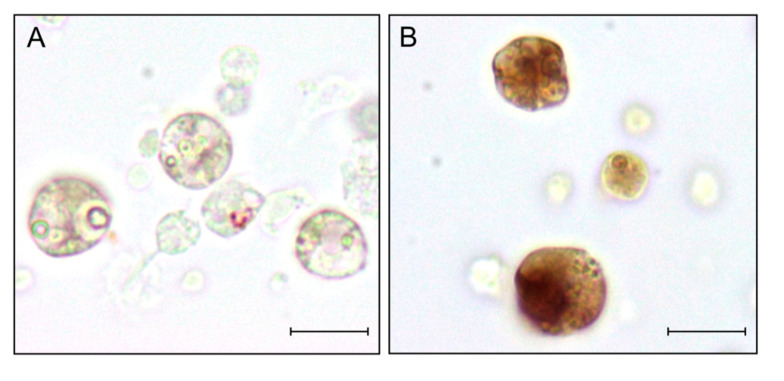
ISH with riboprobes for *bsc3a/c5ar* on hemocyte monolayers. (**A**): sense riboprobe; (**B**): antisense riboprobe. Scale bar: 10 µm.

Only morula cells, and no other immunocytes type, were labelled by the antisense probe for BsC3a/C5aR (Figure 5).

**Figure 6:**
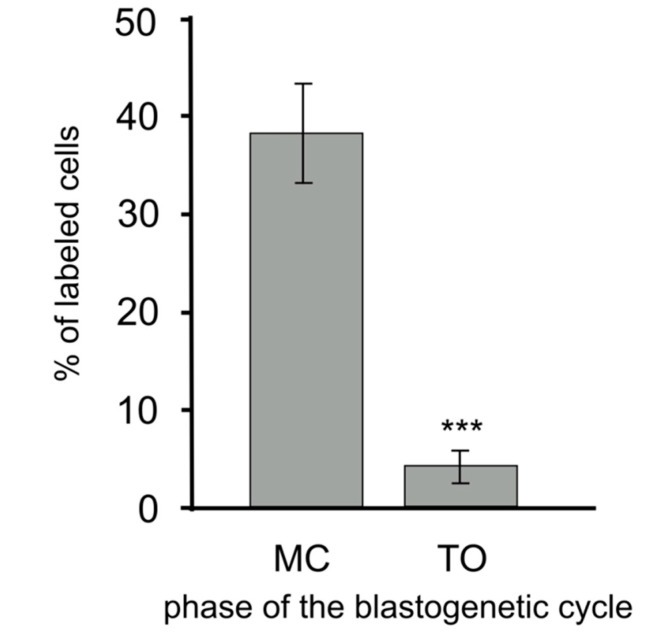
Fraction of hemocytes labeled by the riboprobes for *bsc3a/c5ar* at MC and TO. Asterisks indicate significant differences with respect to the control (***: *p* < 0.001).

Labeled circulating morula cells amounted to almost 38% of the total hemocyte number (about 80% of morula cells) at MC, whereas it significantly (*p* < 0.001) dropped to about 4% of the total circulating cell number (approximately 8% of morula cells; Figure 6).

No variations in the quantity of labeled cells with respect to the control were observed in the cases of zymosan- or *B. clausii*-treated cells (data not shown).

## 4. Discussion

In the present work we identified and characterized a new receptor with similarity with both human C3aR and C5aR receptors, and then named BsC3a/C5aR, from the colonial ascidian *B. schlosseri*. Like the other described C3a and C5a receptors it belongs to the G protein-coupled family of 7TM-domain receptors. Analogously to the vertebrate C3aR, BsC3a/C5aR has a large extracellular loop, between the TM4 and TM5 domains, that, according to Chao et al [10], is important for ligand binding. Indeed, the amino acid sequence alignment with the human orthologue demonstrates that, in this loop, the Tyr corresponding to the position 174 of the human sequence is conserved also in *B. schlosseri* sequence, but not in the sequences of *Ciona* R1 and *Halocynthia*. In humans, this residue plays a critical role in the binding of human C3a and the consequent triggering of signal transduction [10]; the binding is further strengthened by the negatively-charged Asp and Glu residues [34] that are present, also in our case, in the above-cited loop. Therefore, we can argue that, in *Botryllus* as in humans, the second loop is important for ligand binding and the activation of the effector cells. 

Furthermore, the cytosolic C-terminus bears two Thr residues, corresponding to those located at positions 463 and 466 of the human sequence, that represent putative phosphorylation sites and are required for C3aR internalization [35]. The result of multi-alignment indicates that none of the two amino acids are present in the R1 sequence from *C. intestinalis*, whereas, only the second Thr is found in the *Halocynthia* sequence. Receptor internalization is a fast feedback mechanism set up by immunocytes to avoid the deleterious effects of sustained complement activation and the presence of the two key residues reported above suggests that this regulation can take place also in our invertebrate model organism. 

In addition to this putative control mechanism, we also demonstrated, in *Botryllus*, the presence of a positive autocrine feedback loop as the stimulation of BsC3a/C5aR with the C3aR agonist increases the transcription of BsC3 by morula cells. Since the production of C3a depends on the activation of the complement system upon the recognition of nonself, this positive loop can be a mechanism useful to enhance the inflammatory reaction required to kill and clear the foreign material.

In vertebrates, C3a and C5a receptors derive from duplication events which occurred early in their radiation, presumably during the invertebrate-vertebrate transition within chordates [8]. Accordingly, the ascidian C3a/C5aR sequences cluster together as the sister group of the vertebrate C3aR and C5aR clades. 

As an invertebrate, *Botryllus* relies only on innate immunity and circulating immunocytes are responsible for both the cellular immunity and the synthesis and secretion of most of the humoral factors involved in immune responses. It has only two immunocyte types: phagocytes and morula cells. The former can easily ingest foreign cells or particles, especially when opsonized by rhamnose-binding lectin [36] or complement [37], whereas the latter are granular cells involved in inflammatory reactions [22]. Morula cells are the first cells to sense the presence of nonself [24] and, as a consequence of this recognition, they trigger an inflammatory reaction including the selective recruitment of additional morula cells in the infection site, their leakage from the circulation, and their degranulation with the induction of cytotoxicity [36]. Morula cells are also the only cells synthesizing BsC3 upon the recognition of nonself [17,37]. 

In vertebrates, the C3a and C5a receptors are mainly expressed in immunocytes involved in innate immune responses, such as monocytes, macrophages, DCs, neutrophils, basophils, mast cells, and eosinophils, but also in lymphocytes, such as T cells [9]. Here we demonstrate, using ISH, that only morula cells, i.e., the cytotoxic immunocytes able to synthesize BsC3, actively transcribe *bsc3a/c5ar*. This is in agreement with what reported in *C. intestinalis*, where the cells involved in complement-mediated inflammatory reactions are able to synthesize C3aR as well as C3 [14].

In addition, in both qRT PCR and ISH, we observed a significant decrease of *bsc3a/c5ar* transcription during the TO with respect to EC. Since we already reported a significant increase of the amount of *bsc3* transcription at TO with respect to EC [37], the obtained data are indicative of the presence of an additional control against the deleterious effects of prolonged complement activation. It occurs in the form of a negative autocrine feedback loop blocking the transcription of *bsc3a/c5ar* in the presence of a high amount of BsC3 in the circulation so to prevent a diffuse inflammation. Indeed, at the TO, a diffuse apoptosis occurs in tissues of old zooids. Additionally, cells are cleared by circulating phagocytes infiltrating the zooidal tissues without the induction of any inflammatory reaction, which is usually marked by the recruitment and degranulation of morula cells and the induction of cytotoxicity [17].

Further studies are ongoing in our laboratory to better elucidate the relationships between BsC3 and BsC3a/C5aR expression in *B. schlosseri*.

## 5. Conclusions

Collectively, our results demonstrate that a functional C3a/C5aR gene is present in the colonial ascidian *B. schlosseri*. Its amino acid sequence shows various conserved residues in the TM domains, the presence of the residues for binding anaphylatoxin in the second extracellular domain, and of the residues required for receptor internalization in the cytosolic tail. The BsC3a/C5aR gene is actively transcribed by cytotoxic morula cells, the same cells involved in the synthesis and release of BsC3, and its expression is modulated during the colonial blastogenetic cycle. The expression trend and the use of the agonist suggest the presence of positive and negative autocrine loops, the former enhancing the transcription of BsC3 when BsC3a/C5aR is activated by the agonist, the latter decreasing the expression of BsC3a/C5aR at TO when the level of BsC3 mRNA is high.

To our knowledge, this is the first characterization of an invertebrate C3a/C5aR. Further studies are now ongoing to better elucidate the role of BsC3a/C5aR in *Botryllus* immune responses.

## Figures and Tables

**Table 1:** Distribution of exons and their transcript counterparts in the genome and transcriptome sequences of BsC3a/C5aR, respectively.

	Genomic nt Interval	mRNA nt Interval	Length (nt)
Exon 1	1340–1434	1–95	95
Exon 2	2422–2584	96–258	163
Exon 3	2721–2936	259–474	216
Exon 4	4384–4586	475–677	203
Exon 5	4924–5068	678–822	145
Exon 6	5137–5333	823–1019	197

## Supplementary Materials

The following are available online at https://www.mdpi.com/2079-7737/9/9/263/s1, Figure S1. Genomic sequence of BsC3a/C5aR (g020950). Exon sequences are in grey and AG-GT splicing signal consensus sequences are marked in purple. Figure S2. Alignment of ascidian putative anaphylatoxin receptors and human C3aR. The seven TM domains are marked yellow; conserved residues are indicated with an asterisk on the bottom of the alignment; the Tyr and the Thr residues considered important for the binding to anaphylatoxin and receptor internalization, respectively, are marked green and light blue. TM: transmembrane domain; IL: internal loop; EL: external loop; CT: cytosolic terminus. Table S1: Primers used in the various experiments; Table S2: Accession numbers of amino acid sequences of deuterostomes downloaded from databases.
