# Supplementary material for: Insights into the Complement System of Tunicates: C3a/C5aR of the Colonial Ascidian Botryllus schlosseri
Source: Biology (Basel). 2020 Sep 1;9(9):263. doi: 10.3390/biology9090263 (PMC7565592; doi:10.3390/biology9090263)
Supplement: Supplementary file 1 [file biology-09-00263-s001.zip › Supplementary figure 1.docx]

**Supplementary figure 1**. Genomic sequence of BsC3a/C5aR (g020950). Exon sequences are in grey and AG-GT splicing signal consensus sequences are marked in purple.

AGGCTTTTTGTAAGACACCGCTTACGCGGATGAGGTGGCGAGGAGGAGCCTAGGTAAACCTCCAACACTCAGGTATTGGAATAATACGATTGTTGCAATTCGTAGGATCTCACTCATAACAAAATGGAGACCGCAACAGCATGTCATATCGATTCACAGGAATTTATAACTAGTCCGTAAGCAATGTTCCCTCTAAGCTGCGCACATTCGCGTATGCGCAGTGCTGACACGGTCTCTGCGCACAGAAAATCTGAGTTGCGCACAAAAAATTCGGAGTGATGACCTTTTTGTTTAGACGTTACGTACTGTTTTCCAAGGGTCTGCGCAGTGAGTTGGCGAGTGTGCGCGGACTCGTCAAAAATTAGAGGGAACATTGTCCGTAAGGGGTCCCTTGCTCGCAGTCGCTGTTATGGGAGCAATCAGAGAATCTTCCAAATCTAGTCCTTATTCAGCAACATGGACCTAACTGTACCATTTTCAGTCTCATCAGAGGTGCTGGAACATGGAATCATGCCATTTTGTTATTCACAACCAAAACACATGATATATCAAGCACTCAAAAACCGGTATAATTTTATGATGCTACCAGATTCCACTTAGTTTGATGTTGAAGTCAATTATTGCGGGATACGATGGGTTGGAGCATAGTATAAGTTGAAATCCAAGGAATTTGATCATAGATATTAGGTTTAATGGAAAATCCTGGCACTGCACAAAAATGGTCACAGGGTAGAAACTAAAAGGTTCTTTTGCGAGCACGAACCTCTGAATTCTCATGCCTCGCATACCGAACACCAGCGTGAGCCGAACAGTCACACGCTCCAGGAAATTGGACGACGGGACGTATGTGTGTTGGACTTATGCGCTTTAACTTACGAAGTGAAGAATTCGTTGAACATCTCGGGACTGCAACGCAACTATTGCATACACTTGACAATTTCTTGATTCCTAATATCCTGTATATTTAAAACACCAACGTTTTTGAGAAGCGACATCCAGACCGTTTCGTGTTGTTCAAATTTAAAATGTCACATTTTGATATATGCAGAAAATATCGGCGATATCGTCAAAAATATCTTATATCGGCTATAATGGTTTTGGTTCAGAATTCGCCTGGTATATGTCGGGCGCTAATTCCGTCACAGAATAGATGGAATTGCGAGAGAGTGAGGCCGGTACACTTTGTCGTGTATGCTATGTCGTTGACTTAATTCGCTATTCTCCCATCGAGAAATCATGAAGTATAATTCCTGATTATTTTCCAGGTGGCTTTGGCGACCCTGTTGGGCACAATCCCTGCGCGGCAAATCAGATACCTGACGTCGCCATGGCGACCTTTATGAGCTTGGTTGTAGTAGTCGGTATTTTGGGGAATGCCATCGTGTTTTTTGTGATCCTCGTTCTCCAAGAATACCGGAAATCTGTTTCAAATTGGTAAGATTTAGATACAATTATGGCGTGAGAAGTTTATGTATCGTGCTTGATGTGCGAATACATGAATTTAACATGTCGCCGCAGAGCCAATTTAAATTTTTTATTTATTAGGAAACATTCAAAAAAAATTCGAATTTTTATGTCAATCCCATGTAATTGTTGAGTTGTCTTCAAAAGCGATGCACAACAAATTTGACTCAATTCCAACTAGTAGAGACGGAGTTATTTGAGTTTTTCCCGGATTGGTGGATAATTCGAGAATTCAACGAAAAACTCAATTAACTCCGCTTCTACTAGTTGGAATTGAGTCAAATTTGTTTTGCAACCCGGAATCCGGAAAAAAGTTATTTTTGAACAACCCTACAATACAGTCATGTTGTTGGAGTTCATTATCTATGTGGGCAATAGGCACAACGTGTATAATACTAAATAACGCCAGTTACTGATAGCTTAATTGCCTTCGGTAGGCTACTCAGGATGTTCGATTATTATGTAATCCGTATCACCTGCTTTGTCATCTAGATTAGATTCTAGAAACTTGGACTGTAGCCAAATTCTTTCCCGGTAGCTTACAATAGATAGATAACGAATACATGCTTATGAACAGAGATTTCCAAATCAATGATGAAACAACTGCAAAGATCAGGCAATACGTACGTTTGTTAACTTTGTTTTGTGGTATTCATGGTGTTTGTCATTTACACCAGACCGATATTCTTCATTTTTTTCCCGTAACTGCCGACGCACGGCCCACTTCTAGGCTTTTTGTAAGACACCACTTACGCGGATGAAGTGGCGAGGAGGAGCCTGGATAAACCTCCAACACTTAGGTATAGGAATTCTGAAGTCGACCTCGGTCGGACTCGGACCCGCACCCATTGGTGGTGCAGCCGCGGATTGGGCCTATATCATTGCGCTATCTCGTCACCTATATGTAAAGTTGGTGGGATTCTCGGTTAACCGATAAACATTTCTCGCTCATCGTAGGTACGTCCTGCAATTAGCGCTGGCTGATACTCTGTTTCTGCTCATGCTCCCGTTTGGAGCAGCGGAAGAGATGGCGGGGAAATGGTATTTTCCAGCAAGTCTTTGTAAAGCAAAAGAAGGAATTTTGATGGTGAACTACTATGCAAGCATTCTATTCCTCACGGTGAGCCTATAGTATTGATATAATCGCCTGAACTTTGCACTTACGCAACTAAGTGATGGGCGGTGTGTCAGCAGTAACAAATTTATTCTTTCTGCCACATGCTGTAAGTCTACAACCACTTCTGTTGGTGTTTCAGATTATGAGCTTCGACAGATTCGTGGCCGTTACGAGTTCAGGGGTTTCGCGATGGTCGAATGTGCTACGCAGGCTCGACAGTGCGGCACTGATTTCTCTGATTGCTTGGCTCGTGAGCATCGGCTTGGCAGTTCCGATGTACCTCTACAGCCACGTGACACAGTGCGACGAATGCTCCTACAATTTCCCTCTCACTGACCAAGAAAGATGCGATAGGGTAAGCCTTTTGATATAGAAGCATTGTTTTGGGGTAGAGTTAGAGACACCGTAACGCATGTTCAGCCCCCTTGTCAAAACGCCCATATACTTTTGTGATGTGAATAATGTCTTCGTATTAACAGTCACGATTATGAGAATAGTATAATACTATTTCATAAATCTTGAAGCACCAAATTATACTATTGAGACACATTGATTGATGAAGGCTGACTTGATAATGTATAAATTCTTAGACGCATACTGGCAATAATAATATAATGGCTGGTACTGTGAAATAGGTGGAAACAGACCTACTATACCTACTATACTCACACCCGAATCTGGAAGTAAAGCATGAAAGTTAAAATCGCCCCCTAGTCGCCTTTGAGTGCAGGAAAGGCTCGACACTGGTCCACGAAATTTAGTAGGCTCATAAACAGGGTTGAAATGACTCGAGTCCGACTCGCGTCACGATTTTTGATGATTCGACTTGACTCGAGTCATTTTCTCAAAATACGGAAATGACTCGACTCGAGTCAATCTGTGAATTGACTCGACTTGGACTCGAGTCATTGTGCATGACACGGACCAAGTCATATGACTCGAGTCTCAGACGGCCAAGACCGACATTTCATTGTAATTTCGAAGAATAGTAAAACAACCACACTGTTGGCATTTCAGCTATTGGCACTAGTACTTGAAAATGCGACCAGAAACTGTTAAAAGTGAGAAGAATTAGTATATTTTAAATGCGAACCATTCGATATCGCCAAACAGAGTTATAAAAAGTAGTTTCAACAAGACTTTAATTCGAAACTGAGCAGTCAACGCAATGTCTTGGACTCGGACTCGAGTCATAATACTATGACTCGGACTCGACCCTCTGCTCGCTGCTTGGACTCGGACTTGAATCGGTGGTGAATTAAAAAGGACTCGACTTGACTCGAGTCAAATCTATAAATTTGAGATTGACTTGTGACTCGGAGCCTAGTGACTTGGTTACAACCCTGCTCATAAGTATTAAATGTCAATCACGTTTAGTTCCAACCAACTATCATAGGACCATCTTATGTTTCTTGCATATTTAGTGCGAGATTCTAACACTGCATTTAGCCAGAATATAAACCGAAATTAGTATCGTTTACTTGCCTTCTTCATTATTTGTCGTTATCGCGGTGACCCTAAAGCTAAAATTTAAAATGGAAGAAGGTCTGATCGTTAGGACCCTGGCTGATGTGCCAAAAAAGTCATCAAATAACATTTCGTTGATATGACAGCTTGGTTTTCAAATCTATTTAGGATTTTAGTTAAGATCTGGCGTACTCTGGGATTGGTATGGGCTGCATACCGTATAATCGATTAATTTAAACAGTTGTGGGGACGTGTAAAATAGGTAACTATGAGTACCCCGGAAATATGCTGTAATGATGTTGTAAGTTTCAAGTAATGAATTCCATTTCTTGTAGATGTCCTACAACGACACCCAATGTGCAGAATACCTCCTAGAAGAAAGCAATCCAGAAAACGTGTACACCAATTACTTGGAACCCGAAGAATACGACAAATTATTCAATTTTTTCACCCAAATGCAAGACAATATGACCGATGAAAACACCAATACTCTGAAACTACTGAATGAGTTGGCGAATTCCGAAAATGATATTTGCAAGTAAGCCGGAAGAACCGTGACGGAGTGTACTTCTAGTACCCCCTATCGATGTTTTTAAGTTGAAGTCGCGAGTACCAATGATGATGCTTTTATGAATTCGGAATGAGTTCAAATTGTTATGGCTCTTCGTAATCTTCCACAACAGCCATAATAATCACAAAGGCCTACGTCATGACATCCATACACCGATGCCTGATTTTGACGGATACCGGATTTACCAGAAATACGGCATTGCCACGTTGTTCATATCTTGCACACTCAACCAAAAGGTTGCGTACCCCTGGCGATCACATAATTTACGAAGTACATAGATTTCAAAGTCTTCTCTTCTCCCCAGAACGTCTTCGCCGCAGAGTTACCGGACCTGGCTGTATTTGAACGTATCCATTCTCCTAGTTCTGCCGTTTGTGCTTATTTGCATATTTTACGGAATGATTTTATACACCATGATGGGAACTGCAACAACCGCATGCACGAATAAGGTATTATCATTAATGCATTACATCTGTTAAACTGTTGTACACAAATCAACCCAATCCTCTTTTTTCAGCGGCAATATCGTCGACGCGTGACGTTGATGGTGCTAGCGCTCGTCACCTTATTCATCGTTTCTTGGCTGCCTTGGTATGTCGTAACGCTCGCCAAAGTGCGAGGATTCCCCATGTCTGAATCGGGATGTACGAAACTGACCAACTTCGTGCGGGTGCTGACTTATTTGAACAGTGCGCTGAATCCATATTTCTACAGGTGAGATTCATTTTTACAATAGTAGATCTTTGTATGTTTCGTAGGCCCACAATTTACATCAATACCTATTGTGGAGTAAATGTATGAGTAATATACTCTTCTTACGCCATAACCTATTGCGCTGAATGCATGCATATGCTTATCCCGAGTGGACACTGTATTGACAATTAGGCATAGGGGAACGTGCTGAGATAATAAGTTGTTTGATACTTATAATTTATATACGCATCAGCTATATACCATATGATATCTATGACATGGTAGTGATTGATGGCAACCAATGGAAAGTATCCCCGAAACGGAATTGAAACTTCAGCAACGATTATATGATAATTCGCTATTTCCATGCAGTTTACTGAGTACGCGGTTTCCACGGCGGCTGTCCTCTGCGTTCAAAAAGTGTCAGAGATTCTCGTCGACGATCAGATTCGGATCCGGCAGGAAGCGGTCGAGCACAGTGC
