# Supplementary material for: Insights into the Complement System of Tunicates: C3a/C5aR of the Colonial Ascidian Botryllus schlosseri
Source: Biology (Basel). 2020 Sep 1;9(9):263. doi: 10.3390/biology9090263 (PMC7565592; doi:10.3390/biology9090263)
Supplement: Supplementary file 1 [file biology-09-00263-s001.zip › Supplementary figure 2.docx]

**Supplementary figure 2**. Alignment of ascidian putative anaphylatoxin receptors and human C3aR. The seven TM domains are marked yellow; conserved residues are indicated with an asterisk on the bottom of the alignment; the Tyr and the Thr residues considered important for the binding to anaphylatoxin and receptor internalization , respectively, are marked green and light blue. TM: transmembrane domain; IL: internal loop; EL: external loop; CT: cytosolic terminus.

**TM1**

C3aR *Homo sapiens* --------------MAS----FSAETNS---------**TDLLSQPWNEPPVILSMVILSLT 33**

**R1 *Botryllus schlosseri* --------------------------------------------------------MS**LV 4

R *Halocyntia roretzi* MGLLKDMNLTSLYDVESFGDYYYVEPNFTDPM-FFDDMDLCAM--SKPITQPALILTIIV 57

R1 *Ciona intestinalis* ------------------------------------------------MALAQDVVIIFL 12

R3 *Ciona intestinalis* ---------------------------------MISAKDTCPE----PQPV-FAAVAIFF 22

R4 *Ciona intestinalis* -----------MYDLLR-----QLTSNDSGTFTPDQIDNTCDN--PQPNVP-FAVVSVAL 41

**TM2**

**TM1**

**EL1**

**IL1**

C3aR *Homo sapiens* FLLGLPGNGLVLWVAGLKMQRTVNTIWFLHLTLADLLCCLSLPFSLAHLALQ--GQWP-- 89

R1 *Botryllus schlosseri* VVVGILGNAIVFFVILVLQEYRKSVSNWYVLQLALADTLFLLMLPFGAAEE-MAGKWY-- 61

R *Halocyntia roretzi* GLFGLILNFCVISVVAILKEYRISRMHWFFLHMMVADVIFLLFLLILVVNELQ-GEWV-- 114

R1 *Ciona intestinalis* CICGIGLNTAMLGLFCVKKQVRGSIYTTYMIQIAATDLIVCLVWLIMTSLFLHELPTEPT 72

R3 *Ciona intestinalis* VAIGLVGNAIVILLILVLKECSKSITNWYILQLAIADTLFLLMLPFTASSELS-GEWS-- 79

R4 *Ciona intestinalis* VVLGLFGNMVVAFIILVLQEYKKSVANWYILQLALADTLFLLMLPFTASSELV-GQWT-- 98

* * *

**IL2**

**EL1**

**TM3**

C3aR *Homo sapiens* ----------YGRFLCKLIPSIIVLNMFASVFLLTAISLDRCLVVFKPIWCQNH------ 133

R1 *Botryllus schlosseri* ---------FPASLCKAKEGILMVNYYASILFLTIMSFDRSVAVTSSGVS---RWSNVL- 108

R *Halocyntia roretzi* ---------TGFGTCKISEASLFTTHGAGVAFLVMANLSGILSQFKPIKCPLQQLPKFL- 164

R1 *Ciona intestinalis* LQANAEPPPTSSLTLYRFIYAVYNFHYYINIFLLATMSVERCVAVWKSLRCQRYQFNR-- 130

R3 *Ciona intestinalis* ---------YGIVMCKIKEAILFINYYASIYFLVIMSFDRYLAVTKAFSS—SDLVVAL-- 127

R4 *Ciona intestinalis* ---------YGVELCKVKEAILFINYYASIYFLVVMSFDRYVAVTKAFAS—SSLVTRL-- 146

**

**EL2**

**IL2**

**TM4**

C3aR *Homo sapiens* R-NVGMACSICGCIWVVAFVMCIPVFVYREIFTTDNH-NRCGYKFGLSSSLDYPDFYGDP 191

R1 *Botryllus schlosseri* R-RLDSAALISLIAWLVSIGLAVPMYLYSHVTQ--C—-DECSYNFPLT--—DQERC--DR 158

R *Halocyntia roretzi* RKEIVTSLLILAFIWILCSLINLPLYKFSTLN-DC----KHCALEFPV-T-MDEIC---P 214

R1 *Ciona intestinalis* ----KFIYGVSALMWLIAFSCALPSFIRLDLVTDN-INITRPANMKVAETHMYMKCYE-P 184

R3 *Ciona intestinalis* R-GPRAAGIITAIGWVISVGISSPLFIYSTV-GKC--NV-CAYKFPLTKV---ENST--- 176

R4 *Ciona intestinalis* R-SPEASYIFTTAGWIISILFSVPLFMYSSVSG-CH----CAYQFPSYGH—--EYGY--- 194

* *

**EL2**

C3aR *Homo sapiens* LENRS—-LENIVQPPGEMNDRLDPSSFQ—-TN---DHPWTVPTVF-QPQTFQRPSADSLP 243

R1 *Botryllus schlosseri* MS--Y—NDTQCAEESNPENVDK-LFNFFTQ----MY----TNYLEP-EEYNMTDEN---- 201

R *Halocyntia roretzi* MLDF—-NN-T-V-—----CAGL---MKLLFEEEKLANFSETNSSM—-FEEIMQD--DQSG 259

R1 *Ciona intestinalis* LKHASDNNLTYVMDYDGISAVDCYDIMQEDYLSLIDEIDTGMMD-PMYEEYQELYTDDIY 243

R3 *Ciona intestinalis* MM--Y-----GDLYAQMGDAG—--D-P-------M-----T-FL-------GAAANG--- 202

R4 *Ciona intestinalis* M-----------------------------------------------ESYCQQ------ 201

**EL2**

C3aR *Homo sapiens* RGSAR-LT—-SQNLY----SNVFKPADVVSP------------------KI-PSGFPI-- 275

R1 *Botryllus schlosseri* TNT----------LK-LL------------------------------------------ 208

R *Halocyntia roretzi* DD-----------YK—LL—VGQN--------I---------STML------GLTS-C--- 279

R1 *Ciona intestinalis* GGNITMLPELMQFVYQVTLP-NGDNMTVVDHQIYFSLFHGIISHIHNKSKKEPLGGICDP 302

R3 *Ciona intestinalis* -----SAVVPDPINETSIFEDEDFNHQY-------FVDLAEQYGY-------NNY----P 239

R4 *Ciona intestinalis* F---------MQ-------KFPGD---------YESVQHCINETRNTNLT-EHLN—AL-- 232

**EL2**

C3aR *Homo sapiens* EDHETSPLDNSDAFLSTHLKLFPSASSNSFYESELPQGFQDYYNLGQFTDDDQVPTPLVA 335

R1 *Botryllus schlosseri* NEL----A-NSEN---DICKT----SS--------PQ---SYRT---------------- 229

R *Halocyntia_roretzi* -----K----------------PR-S---FYI---------------------------- 286

R1 *Ciona intestinalis* RDTLNK-----DA------KI---------Y----------------------------- 313

R3 *Ciona intestinalis* AVVN---PAASTGVNLNHSTEEFQDFEN-FDENIIP-NTL--IKNMDHTCKHSPNQYYFT 292

R4 *Ciona intestinalis* NES—FPF-DY-DE-FEVFADNITTIAAASE—-TV-TFPGLGNETLIHICKHQENRVFF-V 284

.

**IL3**

**TM5**

C3aR *Homo sapiens* ITITRLVVGFLLPSVIMIACYSFIVFRMQR-------------------GR--------F 368

R1 *Botryllus schlosseri* WLYLNVSILLVLPFLICIFYGMILYTMMGTAT---------------------------- 271

R *Halocyntia roretzi* WLYTTFTFFFLVPLPFLIVYIGLWIYKNKNTEK--------------------------- 319

R1 *Ciona intestinalis* NIIVNLIIGFFIPFLAILISYVSIAHMIRVRALDRMENDCDVIESESTLGRRMSDFLRQF 373

R3 *Ciona intestinalis* WLKVNVSLFFCFPLLAMCCFYGLIIRAIVTTQA------- ------------------- 325

R4 *Ciona intestinalis* WLSVNFVFAFCLPLILISFFYGMIIKTIMQSKK--------------------------- 317

*

**IL3**

C3aR *Homo sapiens* ------------------------------------------------------------ 368

R1 *Botryllus schlosseri* ------------------------------------------------------------ 271

R *Halocyntia roretzi* ------------------------------------------------------------ 319

R1 *Ciona intestinalis* VSGGGPVKNVGESTPPYNKGSSPLLRRSLRKKARDRGTGLEGSEVKSSRDDVVKPPVVRR 433

R3 *Ciona intestinalis* -------------------------------------------- ---------------- 325

R4 *Ciona intestinalis* ------------------------------------------------------------ 317

**IL3**

C3aR *Homo sapiens* ------------------------------------------------------------ 368

R1 *Botryllus schlosseri* ------------------------------------------------------------ 271

R *Halocyntia roretzi* ------------------------------------------------------------ 319

R1 *Ciona intestinalis* EEKPVVRDAGVDRRPRLSSFSHPTHSRRTSGGSASSAESFQTDQTSLHAATPSPGVRLSA 493

R3 *Ciona intestinalis* ------------------------------------------------------------ 325

R4 *Ciona intestinalis* ---------------------------------------FQ---YN-------------- 321

**IL3**

C3aR *Homo sapiens* ------------------------------------------------------------ 368

R1 *Botryllus schlosseri* ------------------------------------------------------------ 271

R *Halocyntia roretzi* ------------------------------------------------------------ 319

R1 *Ciona intestinalis* PIFVHDKTPVQRASSTETPTHPRSPPTRRWSSETLRSPHEASPPVLDETEPLYDRVDDVT 553

R3 *Ciona intestinalis* ------------------------------------------------------------ 325

R4 *Ciona intestinalis* ------------------------------------------------------------ 321

**IL3**

C3aR *Homo sapiens* ------------------------------------------------------------ 368

R1 *Botryllus schlosseri* ------------------------------------------------------------ 271

R *Halocyntia roretzi* ------------------------------------------------------------ 319

R1 *Ciona intestinalis* MAKPTSPPSGDSTQELFHGSYARLYSQKPDARPRYQRQASTTSNYRSRAYSATSRAGGGG 613

R3 *Ciona intestinalis* ------------------------------------------------------------ 325

R4 *Ciona intestinalis* ------------------------------------------------------------ 321

**EL3**

**TM6**

**IL3**

**TM7**

C3aR *Homo sapiens* ----AKSQSK-------TFRVAVVVVAVFLVCWTPYHIFGVLSLLTDPETPLGKTLMSWD 417

R1 *Botryllus schlosseri* -----TACTNKRQYRRRVTLMVLALVTLFIVSWLPWYVVTLAKVRGFPMS-E-SGCTKLT 324

R *Halocyntia roretzi* -----IKNNSKPKDL--PANVIAATSILFILCWAPWNSVQLRKVNGIAHE-EGSYCENLA 371

R1 *Ciona intestinalis* GTLRKTIVHKNAERHLRISRTALTYVCVFAVCWLPQRIVAVVYVVEGMVGLGGHACHAVF 673

R3 *Ciona intestinalis* ---GAGRNEVQKY-RNRVTVIVLALICLFLVSWLPWYTVQLALMNGISLS-N-SECKRLT 379

R4 *Ciona intestinalis* ----VGNNEAQRSYRRRVTTIVLALVFLFVASWLPWHSFQLAKIVGFPMPAE-S-CTNFQ 375

* *

**CT**

**TM7**

C3aR *Homo sapiens* HVCIALASANSCFNPFLYALLGKDFRKKARQSIQGILE------ ---------------- 455

R1 *Botryllus schlosseri* NFVRVLTYLNSALNPYFYSFMGSRFHRRFRR-ARSTATRKYRFLS-VLSRLSFHG--QDS 380

R *Halocyntia roretzi* SYSRLIAICYNAVLPLVYIILTPDFRFRTLS-AIATACQYFPTI-WYK-DKSNS-----S 423

R1 *Ciona intestinalis* TATRILSFLSVLLNPIVYAMTQREIRHL-LRRKLAGMCKCIHPGR------------E-- 718

R3 *Ciona intestinalis* YAVRLIAYLNSTLNPYFYGTLGGRFAER-LRKARSKLGFVYT----LSITSDTRRRARSW 444

R4 *Ciona intestinalis* YGVRITAYLSSALNPFLYSFLGARFAQR-LTKAKETMRLSATRHSRVLSGDSKRNKRHH- 433

*

**CT**

C3aR *Homo sapiens* -----------------------------------AAFSEELTRSTHC-----PSN--NV 473

R1 *Botryllus schlosseri* NNSTSGGTCKTNLTGRDLKLSTRI-KSNSDDDSRSSVR----TERTVVDQTHLPS---VT 431

R *Halocyntia roretzi* QNSEGKIETSDCEMVNCELPASSDDKSQKTDLL----------PDTNV------------ 460

R1 *Ciona intestinalis* ------------------------------------SNPDPQNRDVNVSFANARRDFSVV 742

R3 *Ciona intestinalis* NN-SARM-AN--GLKDQDAPSGRLHIVENGKLS-----MGQRTERTAV------------ 483

R4 *Ciona intestinalis* STSHSGSVRQPTAERPAEAARDVVYWRGTTENGNTGETFANKTEKTEV------------ 485

**CT**

C3aR *Homo sapiens* ISERN-STTV-------------------------------------------------- 482

R1 *Botryllus schlosseri* -S--T-------------- ---------------------------------------- 422

R *Halocyntia roretzi* ---------------- ------------------------------------------- 460

R1 *Ciona intestinalis* AHNITSDEIFMKRERHARNKERWSRLMHRLGGKNLRLSLTGSGFTSPGEKQSDGTIDEDE 802

R3 *Ciona intestinalis* ----------- ------------------------------------------------ 483

R4 *Ciona intestinalis* ----------- ------------------------------------------------ 485

**CT**

C3aR *Homo sapiens* ---------------------------------------------- 482

R1 *Botryllus schlosseri* ---------------------------------------------- 431

R *Halocyntia roretzi* ---------------------------------------------- 460

R1 *Ciona intestinalis* EDKGQSSKGQSSSTSFKLIGGWEDCQQMIDMAEVNKGQTTNEETIL 848

R3 *Ciona intestinalis* ---------------------------------------------- 483

R4 *Ciona intestinalis* ---------------------------------------------- 485
