# Supplementary material for: Insights into the Complement System of Tunicates: C3a/C5aR of the Colonial Ascidian Botryllus schlosseri
Source: Biology (Basel). 2020 Sep 1;9(9):263. doi: 10.3390/biology9090263 (PMC7565592; doi:10.3390/biology9090263)
Supplement: Supplementary file 1 [file biology-09-00263-s001.zip › Supplementary table 2.docx]

**Supplementary table 2**. Accession numbers of amino acid sequences of deuterostomes downloaded from databases

| **Protein** | **Specie** | **Accession numbers** | **Database** |
| --- | --- | --- | --- |
| C3aR | *Homo sapiens* | NP_004045.1 | Genbank |
| C3aR | *Pongo abelii* | NP_001124844.1 | Genbank |
| C3aR | *Mus musculus* | NP_033909.1 | Genbank |
| C3aR | *Bos taurus* | NP_001077221.1 | Genbank |
| C3aR | *Pteropus alecto* | XP_006914735.1 | Genbank |
| C3aR | *Myotis lucifugus* | XP_006084265.1 | Genbank |
| C3aR | *Erinaceus europaeus* | XP_007537599.1 | Genbank |
| C3aR | *Monodelphis domestica* | XP_001367766.1 | Genbank |
| C3aR | *Gallus gallus* | NP_001025940.1 | Genbank |
| C3aR | *Meleagris gallopavo* | XP_003202599.1 | Genbank |
| C3aR | *Cuculus canorus* | XP_009560745.1 | Genbank |
| C3aR | *Columba livia* | XP_005509824.1 | Genbank |
| C3aR | *Serinus canaria* | XP_009083801.1 | Genbank |
| C3aR | *Falco peregrinus* | XP_005234503.1 | Genbank |
| C3aR | *Struthio camelus australis* | XP_009680583.1 | Genbank |
| C3aR | *Aptenodytes forsteri* | XP_009289203.1 | Genbank |
| C3aR | *Geospiza fortis* | XP_005427462.1 | Genbank |
| C3aR | *Alligator sinensis* | XP_006032697.1 | Genbank |
| C3aR | *Alligator mississippiensis* | XP_006267655.1 | Genbank |
| C3aR | *Anolis carolinensis* | XP_003222918.1 | Genbank |
| C3aR | *Thamnophis sirtalis* | XP_013931138.1 | Genbank |
| C3aR | *Python bivittatus* | XP_007436373.1 | Genbank |
| C3aR | *Chelonia mydas* | XP_007055687.1 | Genbank |
| C3aR | *Xenopus laevis* | NP_001267568.1 | Genbank |
| C3aR | *Xenopus tropicalis* | XP_002941252.1 | Genbank |
| C3aR | *Danio rerio* | XP_009303337.1 | Genbank |
| C3aR | *Oncorhynchus mykiss* | NP_001117875.1 | Genbank |
| C3aR | *Lepisosteus oculatus* | XP_006627133.1 | Genbank |
| C3aR | *Salmo salar* | XP_013994139.1 | Genbank |
| C3aR | *Esox lucius* | XP_010901409.1 | Genbank |
| C3aR | *Callorhinchus milii* | XP_007884079.1 | Genbank |
| R1 | *Ciona intestinalis* | CAI84650.1 | Genbank |
| R2 | *Ciona intestinalis* | XP_018672649 | Genbank |
| R3 | *Ciona intestinalis* | XP_004226218.2 | Genbank |
| R4 | *Ciona intestinalis* | XM_002121537.3 | Genbank |
| R | *Phallusia mammillata* | LR786768.1 | Genbank |
| R1 | *Botryllus schlosseri* | MN053062 | Genbank |
| R2 | *Botryllus schlosseri* | g25017.01 | Aniseed |
| R3 | *Botryllus schlosseri* | g29610.01 | Aniseed |
| R | *Halocyntia roretzi* | S88.g02174.01 | Aniseed |
| C5aR | *Homo sapiens* | NP_001727.1 | Genbank |
| C5aR | *Gorilla gorilla* | CAA66317.1 | Genbank |
| C5aR | *Chlorocebus sabaeus* | XP_007995541.1 | Genbank |
| C5aR | *Colobus angolensis palliatus* | XP_011782107.1 | Genbank |
| C5aR | *Callithrix jacchus* | XP_008986532.1 | Genbank |
| C5aR | *Oryctolagus cuniculus* | XP_002722914.1 | Genbank |
| C5aR | *Bos taurus* | NP_001007811.2 | Genbank |
| C5aR | *Felis catus* | XP_006941142.1 | Genbank |
| C5aR | *Mus musculus* | NP_031603.2 | Genbank |
| C5aR | *Rattus norvegicus* | NP_446071.1 | Genbank |
| C5aR | *Alligator mississippiensis* | XP_006277268.2 | Genbank |
| C5aR | *Struthio camelus australis* | XP_009668587.1 | Genbank |
| C5aR | *Columba livia* | XP_005515722.1 | Genbank |
| C5aR | *Latimeria chalumnae* | XP_005996096.1 | Genbank |
| C5aR | *Anolis carolinensis* | XP_003222918.1 | Genbank |
| C5aR | *Xenopus tropicalis* | XP_002941328.1 | Genbank |
| C5aR | *Gallus gallus* | XP_015128540.1 | Genbank |
| FPR1 | *Homo sapiens* | P21462.3 | Genbank |
| FMLPR | *Homo sapiens* | NP_002020.1 | Genbank |
| FMLPR | *Mus musculus* | NP_038549.1 | Genbank |
| FPRL1 | *Mus musculus* | NP_032068.2 | Genbank |
