# Supplementary material for: Insights into the Complement System of Tunicates: C3a/C5aR of the Colonial Ascidian Botryllus schlosseri
Source: Biology (Basel). 2020 Sep 1;9(9):263. doi: 10.3390/biology9090263 (PMC7565592; doi:10.3390/biology9090263)
Supplement: Supplementary file 1 [file biology-09-00263-s001.zip › Supplementary table 1.docx]

**Supplementary table 1**. Primers used in the various experiments

| Transcript | Name | 5’-> 3’ |
| --- | --- | --- |
| BsC3aR | BsC3aR-F1  BsC3aR-R1  BsC3aR-F-rt  BsC3aR-R-rt | AGATGGCGGGGAAATGGTAT  GGTCAGTTTCGTACATCCCG  CTGATTGCTTGGCTCGTGAG  CATTGGGTGTCGTTGTAGGA |
| BsC3 | BsC3-F-rt  BsC3-R-rt | ACAAACAGGACCCGAACAAG  TGAGCGCCACATACTGTCTT |
| BsEF | BsEFF  BsEFR | GCCGCCATACTCTGAAGC  GTCCAACTGGCACTGTTCC |
| dT-anchor |  | GCAGTGGTAACAACGCAGAGTTTTTTTTTTTTTTTTTTTTT |
